# Gut commensal microbiota and decreased risk for *Enterobacteriaceae* bacteriuria and urinary tract infection

**DOI:** 10.1080/19490976.2020.1805281

**Published:** 2020-08-30

**Authors:** Matthew Magruder, Emmanuel Edusei, Lisa Zhang, Shady Albakry, Michael J. Satlin, Lars F. Westblade, Line Malha, Christina Sze, Michelle Lubetzky, Darshana M. Dadhania, John R. Lee

**Affiliations:** a Division of Nephrology and Hypertension, Department of Medicine, Weill Cornell Medicine, New York, NY, USA; b Division of Infectious Diseases, Department of Medicine, Weill Cornell Medicine, New York, NY, USA; c Department of Pathology and Laboratory Medicine, Weill Cornell Medicine, New York, NY, USA; d Department of Urology, NewYork Presbyterian Hospital – Weill Cornell Medical Center, New York, NY, USA; e Department of Transplantation Medicine, New York Presbyterian Hospital – Weill Cornell Medical Center, New York, NY, USA

**Keywords:** Microbiota, bacteriuria, urinary tract infection, *Enterobacteriaceae*, *Faecalibacterium*, *Romboutsia*, *Lactobacillus*

## Abstract

Urinary tract infection (UTI) is a common complication in kidney transplant recipients and can lead to significant morbidity and mortality. Recent evidence supports a role for the gut as a source for UTIs but little is known about the relationship between gut commensal bacteria and UTI development. We hypothesized that the abundance of gut commensal bacteria is associated with a lower risk of developing bacteriuria and UTIs. We performed gut microbiome profiling using 16S rRNA gene sequencing of the V4-V5 hypervariable region on 510 fecal specimens in 168 kidney transplant recipients. Fifty-one kidney transplant recipients (30%) developed *Enterobacteriaceae* bacteriuria within the first 6 months after transplantation (*Enterobacteriaceae* Bacteriuria Group) and 117 did not (No *Enterobacteriaceae* Bacteriuria Group). The relative abundances of *Faecalibacterium* and *Romboutsia* were significantly higher in the fecal specimens from the No *Enterobacteriaceae* Bacteriuria Group than those from the *Enterobacteriaceae* Bacteriuria Group (Adjusted *P* value<.01). The combined relative abundance of *Faecalibacterium* and *Romboutsia* was inversely correlated with the relative abundance of *Enterobacteriaceae* (r = −0.13, *P* = .003). In a multivariable Cox Regression, a top tercile cutoff of the combined relative abundance of *Faecalibacterium* and *Romboutsia* of ≥13.7% was independently associated with a decreased risk for *Enterobacteriaceae* bacteriuria (hazard ratio 0.3, *P* = .02) and *Enterobacteriaceae* UTI (hazard ratio 0.4, *P* = .09). In conclusion, we identify bacterial taxa associated with decreased risk for *Enterobacteriaceae* bacteriuria and *Enterobacteriaceae* UTI in kidney transplant recipients, which supports future studies on modulating the gut microbiota as a novel treatment for preventing UTIs.

## Introduction

Kidney transplant recipients have increased survival and improved quality of life compared to patients with end-stage renal disease who receive renal replacement therapies.^1^ However, the immunosuppressive medications used to prevent rejection of the transplanted kidney leads to an immunocompromised state and frequent infectious complications.^2^ Urinary tract infection (UTI) is the most common infection in kidney transplant recipients,^2,3^ affecting approximately 20% in the first 3 months after transplantation with *Enterobacteriaceae* being the most common cause of post-transplant UTI.^4^ In severe cases, UTI can lead to urosepsis, allograft damage, and mortality in this immunosuppressed population.^5-7^ Asymptomatic bacteriuria, a positive urine culture without associated symptoms, is also very common and has been associated with development of pyelonephritis and allograft damage.^6^ Many kidney transplant recipients unfortunately develop recurrent UTIs, which further increase the risk for allograft failure and mortality.^8^ Prevention of UTI is thus an important unmet need in kidney transplant recipients.

Recent studies support a role for the gut microbiota in the pathogenesis of UTI.^9-11^ An elegant review by Flores-Mireles et al. reports the first step in the development of UTI as contamination of the peri-urethral space with gut bacteria.^12^ In a study of non-transplant patients, Paalanne and colleagues found that the gut abundance of *E. coli* was higher in children with *E. coli* UTI than in children without *E. coli* UTI^10^, suggesting a role of the gut microbiota in UTI development. In a study of 168 kidney transplant recipients at our center, we found that the gut abundance of *Escherichia* was associated with future development of *Escherichia* bacteriuria and UTI.^9^ We further established at the strain level that the *E. coli* in the urine was genetically most similar to the *E. coli* in the fecal specimens from the same subject, supporting the concept that the gut is a primary source of UTI.^9^ Targeting the gut microbiota to modify the risk for UTI development is particularly attractive as currently known clinical risk factors for post-transplant UTI such as gender and age^4^ are not modifiable. Indeed, recent case reports have reported that fecal microbial transplantation (FMT) is associated with decreased UTI recurrence.^13,14^ No study, however, has evaluated the relationship between commensal gut bacteria and UTI development and such a study would help to identify important bacterial taxa that could be beneficial for modifying the risk for UTI development.

In the current study, we evaluated the microbial profiles previously characterized in our study of 168 kidney transplant recipients to assess the relationship between the relative abundance of commensal bacterial taxa and the development of *Enterobacteriaceae* bacteriuria and UTI. We report that the combined relative abundance of *Faecalibacterium* and *Romboutsia* is inversely associated with the relative abundance of *Enterobacteriaceae* and is associated with a decreased risk for *Enterobacteriaceae* bacteriuria and UTI.

## Results

### Characteristics of the transplant cohort

One hundred sixty-eight kidney transplant recipients provided 510 fecal specimens within the first 3 months after kidney transplantation. Fifty-one (30%) kidney transplant recipients developed *Enterobacteriaceae* bacteriuria within the first 6 months after transplantation (*Enterobacteriaceae* Bacteriuria Group) and 117 did not (No *Enterobacteriaceae* Bacteriuria Group). The *Enterobacteriaceae* bacteriuria cases included: 126 *E. coli* bacteriuria episodes from 36 kidney transplant recipients; 46 *Klebsiella* species bacteriuria episodes from 20 kidney transplant recipients; 9 *Enterobacter cloacae* bacteriuria episodes from 2 kidney transplant recipients; and 3 *Citrobacter freundii* bacteriuria episodes from 3 kidney transplant recipients; and 1 *Raoultella ornithinolytica* bacteriuria episode from 1 kidney transplant recipient. The median time to the development of first *Enterobacteriaceae* bacteriuria was 28 days with an interquartile range of 9 to 83. Among the 51 kidney transplant recipients who developed *Enterobacteriaceae* bacteriuria, 37 (73%) had more than one *Enterobacteriaceae* bacteriuria within 6 months post-transplantation, and 33 (65%) developed *Enterobacteriaceae* UTI with 9 (18%) who had more than one *Enterobacteriaceae* UTI within 6 months post-transplantation. The median time to the development of first *Enterobacteriaceae* UTI was 45 days with an interquartile range of 13 to 89.

We compared the demographical and transplant characteristics between the *Enterobacteriaceae* Bacteriuria Group and the No *Enterobacteriaceae* Bacteriuria Group (Table 1). Female gender was significantly more common in the *Enterobacteriaceae* Bacteriuria Group than in the No *Enterobacteriaceae* Bacteriuria Group (73% vs 33%, respectively, *P* < .001, Fisher’s exact test) and cefazolin preoperative prophylaxis was significantly less common in the *Enterobacteriaceae* Bacteriuria Group than in the No *Enterobacteriaceae* Bacteriuria Group (73% vs 87%, respectively, *P* = .03, Fisher’s exact test). Age, African-American race, history of diabetes mellitus, cause of end-stage renal disease, panel reactive antibody status, deceased donor transplantation, delayed graft function, trimethoprim-sulfamethoxazole prophylaxis, anti-thymocyte globulin induction therapy, and prednisone maintenance were not significantly different between the two groups (*P* > .05, Wilcoxon rank sum test or Fisher’s exact test).

**Table 1.** **Clinical Characteristics in the No *Enterobacteriaceae* Bacteriuria Group and the *Enterobacteriaceae* Bacteriuria Group**. *P* values were calculated using the Fisher’s exact test for dichotomous values and using the Wilcoxon rank sum test for continuous variables. ESRD, end-stage renal disease; DM, diabetes mellitus; HTN, hypertension; PRA panel reactive antibody; PCP, *Pneumocystis jiroveci.*

	*Enterobacteriaceae*	No *Enterobacteriaceae*	
	Bacteriuria Group	Bacteriuria Group	
	(N = 51)	(N = 117)	
Characteristic	N (%) or median	N (%) or median	P value
Age, Years	57	53	0.29
Female Gender	37 (73%)	39 (33%)	3.7 x 10^−6^
African American Race	12 (24%)	32 (27%)	0.70
History of Diabetes Mellitus	18 (35%)	31 (26%)	0.27
Cause of ESRD – DM	18 (35%)	30 (26%)	0.26
Cause of ESRD – HTN	7 (14%)	20 (17%)	0.65
PRA ≥ 80%	5 (10%)	8 (7%)	0.54
Decreased Donor Transplantation	17 (33%)	32 (27%)	0.46
Delayed Graft Function	10 (20%)	18 (15%)	0.51
Cefazolin Preoperative Abx	37 (73%)	102 (87%)	0.03
Trimethoprim/Sulfamethoxazole PCP Prophylaxis	50 (98%)	109 (93%)	0.28
Anti-thymocyte Globulin Induction	39 (76%)	89 (76%)	0.99
Prednisone Maintenance	17 (33%)	28 (24%)	0.26

### *Gut microbial composition associated with* Enterobacteriaceae *bacteriuria*

Microbiome profiling was previously performed on each of the 510 fecal specimens from the 168 kidney transplant recipients using 16S rRNA gene sequencing of the V4-V5 hypervariable region.^9^ The median number of bacterial sequences was 15,841 with an interquartile range of 11,262 to 22,510.

**Figure 1.**
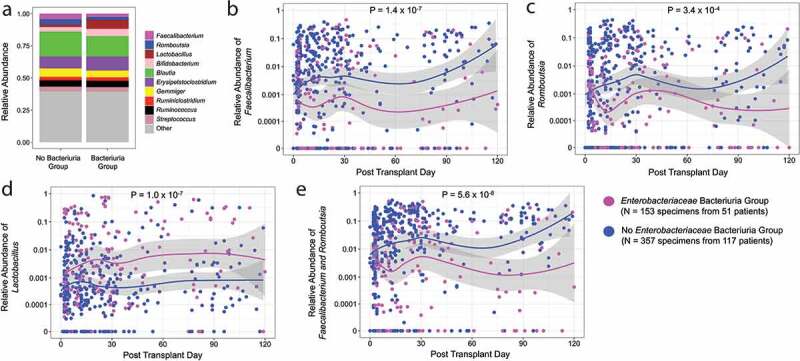
**Relative abundances of the most common Genera by *Enterobacteriaceae* Bacteriuria Group Status. Panel a**. The mean relative abundances of the 10 most common genera are represented on the y axis by color and the *Enterobacteriaceae* Bacteriuria Group status is on the x axis. The *Enterobacteriaceae* Bacteriuria Group consisted of 153 fecal specimens from 51 patients and the No *Enterobacteriaceae* Bacteriuria Group consisted of 357 fecal specimens from 117 patients. **Panels b – e**. The 510 fecal specimens are shown in each graph and each point represents a fecal specimen and the point’s color represents *Enterobacteriaceae* Bacteriuria Group status. The relative abundance of genera is on the y axis (log_10_ scale) and the post-transplant day is on the x axis. The line represents a locally estimated scatterplot smoothing curve with 95% confidence intervals in the shaded area. *P* values were calculated using the Wilcoxon rank sum test. **Panel b**. Relative abundance of *Faecalibacterium* is shown with 131 fecal specimens having a zero relative abundance. **Panel c**. Relative abundance of *Romboutsia* is shown with 129 specimens having a zero relative abundance. **Panel d**. Relative abundance of *Lactobacillus* is shown with 61 specimens having a zero relative abundance. **Panel e**. Combined relative abundance of *Faecalibacterium* and *Romboutsia* with 60 specimens having a zero relative abundance.

Because we wanted to identify the most common bacterial taxa whose relative abundances were significantly associated with *Enterobacteriaceae* bacteriuria, we compared the relative abundances of the top 10 most common genera in the cohort between the 153 fecal specimens from the 51 patients in the *Enterobacteriaceae* Bacteriuria Group and the 357 fecal specimens from the 117 patients in the No *Enterobacteriaceae* Bacteriuria Group (Figure 1a). The relative abundances of *Faecalibacterium* and *Romboutsia* were significantly higher in the No *Enterobacteriaceae* Bacteriuria Group than in the *Enterobacteriaceae* Bacteriuria Group and the relative abundance of *Lactobacillus* was significantly lower in the No *Enterobacteriaceae* Bacteriuria Group than in the *Enterobacteriaceae* Bacteriuria Group (Adjusted *P* < .01, Wilcoxon rank sum test, Bonferroni adjustment for multiple hypothesis testing) (Table 2). The relative abundance of *Faecalibacterium*, the relative abundance of *Romboutsia*, the relative abundance of *Lactobacillus*, and the combined relative abundance of *Faecalibacterium* and *Romboutsia* are shown over time (Figure 1b-1e). The combined relative abundance of *Faecalibacterium* and *Romboutsia* was significantly higher in the No *Enterobacteriaceae* Bacteriuria Group than in the *Enterobacteriaceae* Bacteriuria Group (median 4.0% vs. 0.7%, respectively, *P* < .001, Wilcoxon rank sum test). We also evaluated the subset of 33 kidney transplant recipients with *Enterobacteriaceae* UTI among the *Enterobacteriaceae* Bacteriuria Group. The combined relative abundance of *Faecalibacterium* and *Romboutsia* was significantly higher in the No *Enterobacteriaceae* Bacteriuria Group than in the *Enterobacteriaceae* UTI Group (median 4.0% vs. 1.1%, respectively, *P* value < .001, Wilcoxon rank sum test).

Because female gender was strongly associated with *Enterobacteriaceae* bacteriuria, we evaluated whether there were differences in the relative abundances of *Faecalibacterium, Romboutsia, Lactobacillus*, and the combined relative abundance of *Faecalibacterium* and *Romboutsia* between female patients and male patients. The relative abundance of *Faecalibacterium, Romboutsia*, and the combined relative abundance of *Faecalibacterium and Romboutsia* were significantly lower in the fecal specimens from female patients than in those from male patients (*P* = .003, *P* = .03, *P* = .004, Wilcoxon rank sum test, respectively) and the relative abundance of *Lactobacillus* was significantly higher in the fecal specimens from female patients in those from male patients (*P* < .001) (Supplementary Figure 1). We also evaluated whether the relative abundances of these taxa changed after diagnosis of first *Enterobacteriaceae* bacteriuria or *Enterobacteriaceae* UTI. The relative abundance of *Faecalibacterium, Romboutsia, Lactobacillus*, and the combined relative abundance of *Faecalibacterium* and *Romboutsia* did not significantly change from the closest specimen prior to diagnosis of first *Enterobacteriaceae* bacteriuria to the first specimen after *Enterobacteriaceae* bacteriuria diagnosis (N = 24 patients, *P* > .05, Wilcoxon signed-rank test) (Supplementary Figure 2) or from the closest specimen prior to diagnosis of first *Enterobacteriaceae* UTI to the first specimen after *Enterobacteriaceae* UTI diagnosis (N = 15 patients, *P* > .05) (Supplementary Figure 3).

### The relative abundances of Faecalibacterium, Romboutsia, and Lactobacillus are correlated with relative abundance of Enterobacteriaceae and gut microbial diversity

We next evaluated the relationship between the relative abundance of *Faecalibacterium* and *Romboutsia* and the relative abundance of *Enterobacteriaceae*. The relative abundance of *Faecalibacterium* (r = −0.09, *P* = .04, Pearson’s correlation), the relative abundance of *Romboutsia* (r = −0.10, *P* = .02), and the combined relative abundance of *Faecalibacterium* and *Romboutsia* (r = −0.13, *P* = .003) were inversely correlated to the relative abundance of *Enterobacteriaceae*. The relative abundance of *Lactobacillus* was not correlated with the relative abundance of *Enterobacteriaceae* (r = 0.05, *P* = .29). The relative abundance of *Lactobacillus* was inversely correlated with the combined relative abundance of *Faecalibacterium* and *Romboutsia* (r = −0.15, *P* = 4.5 x 10^−4^). Taken together with our findings that the gut abundance of uropathogens (e.g. *E*. coli) is associated with future development of bacteriuria,^9^ our data suggest that the inverse relationship between the relative abundances of *Faecalibacterium* and *Romboutsia* and relative abundance of *Enterobacteriaceae* could be a factor associated with bacteriuria and UTI development.

We further evaluated whether the relative abundances of *Faecalibacterium* and *Romboutsia* were associated with microbial diversity as measured by Shannon diversity index. The relative abundance of *Faecalibacterium* (r = 0.16, *P* = 4.0 x 10^−4^), the relative abundance of *Romboutsia* (r = 0.08, *P* = .07), and the combined relative abundance of *Faecalibacterium* and *Romboutsia* (r = 0.16, *P* = 3.0 x 10^−4^) were positively correlated with the Shannon diversity index and the relative abundance of *Lactobacillus* was negatively correlated with the Shannon diversity index (r = −0.24, *P* = 2.8 x 10^−8^). The positive correlation between the relative abundances of the two genera and the relative abundance of *Enterobacteriaceae* supports *Faecalibacterium* and *Romboutsia* as markers of gut microbial diversity.

### Antibiotic administration is associated with relative abundances of Faecalibacterium, Romboutsia, and Lactobacillus

Given that antibiotics alter the gut microbiota,^15,16^ we evaluated the relationship between antibiotic administration and the relative abundances of *Faecalibacterium, Romboutsia*, and *Lactobacillus*. In the cohort, 89 kidney transplant recipients (53%) received additional antibiotics aside from surgical antibiotic prophylaxis and *Pneumocystis jiroveci* prophylaxis within the first 3 months after transplantation (Abx Group) and 79 did not (No Abx Group). These additional antibiotics were used for: presumed or documented infection in 50 patients (56%) with the most common documented infection being bacteriuria or UTI; antibiotic prophylaxis in 38 patients (43%) with the most common indication being ureteral stent removal; and gastric motility in 1 patient (1%). Among the 89 patients who had antibiotic exposure, 69 (78%) had a stool specimen before first antibiotic exposure. The relative abundance of *Faecalibacterium* was significantly higher in the 235 fecal specimens from the No Abx Group than in the 275 fecal specimens from the Abx Group (median 1.5% vs. 0.1%, *P* < .001, Wilcoxon rank sum test); the relative abundance of *Romboutsia* was significantly higher in the 235 fecal specimens from the No Abx Group than in the 275 fecal specimens from the Abx Group (median 1.3% vs. 0.1%, *P* < .001, Wilcoxon rank sum test); the relative abundance of *Lactobacillus* was significantly lower in the 235 fecal specimens from the No Abx Group than in the 275 specimens from the Abx Group (median 0.04% vs. 0.1%, *P* < .001, Wilcoxon rank sum test); the combined relative abundance of *Faecalibacterium* and *Romboutsia* was significantly higher in the 235 fecal specimens from the No Abx Group than in the 275 fecal specimens from the Abx Group (median 6.9% vs. 0.8%, *P* < .001, Wilcoxon rank sum test). The relative abundance of *Faecalibacterium*, the relative abundance of *Romboutsia*, the relative abundance of *Lactobacillus*, and the combined relative abundance of *Faecalibacterium* and *Romboutsia* are shown over time (Supplemental Figure 4). Our data reveals that antibiotic administration is associated with lower relative abundance of *Faecalibacterium* and *Romboutsia* and higher relative abundance of *Lactobacillus.*

We further analyzed the relationship between antibiotic administration and the relative abundances of *Faecalibacterium* and *Romboutsia* by antibiotic subgroup. The most common antibiotics used in the cohort were: beta-lactams (Beta-lactam Group, N = 56 patients) and fluoroquinolones (Fluoroquinolone Group, N = 51 patients). The relative abundance of *Faecalibacterium*, the relative abundance of *Romboutsia*, and the combined relative abundance of *Faecalibacterium* and *Romboutsia* were all significantly higher in the 235 fecal specimens from the No Abx Group than in the 177 fecal specimens from the Beta-lactam Group and the relative abundance of *Lactobacillus* was significantly lower in the 235 fecal specimens from the No Abx Group than in the 177 fecal specimens from the Beta-lactam Group (*P* < .001, respectively, Wilcoxon rank sum test). The relative abundance of *Faecalibacterium*, the relative abundance of *Romboutsia*, and the combined relative abundance of *Faecalibacterium* and *Romboutsia* were all significantly higher in the 235 fecal specimens from the No Abx Group than in the 154 fecal specimens from the Fluoroquinolone Group (*P* < .001, respectively, Wilcoxon rank sum test) and the relative abundance of *Lactobacillus* was significantly lower in the 235 fecal specimens from the No Abx Group than in the 154 fecal specimens from the Flouroquinolone Group (*P* = .005, Wilcoxon rank sum test).

### The combined relative abundance of Faecalibacterium and Romboutsia is associated with a decreased risk for Enterobacteriaceae bacteriuria and UTI

We next evaluated whether the relative abundances of the identified genera are associated with a decreased risk for future development of *Enterobacteriaceae* bacteriuria and UTI. We utilized a time-dependent Cox regression to evaluate for the future risk of both *Enterobacteriaceae* bacteriuria and UTI. As a cutoff for relative abundance, we utilized the top tercile cutoff where at least a third of the kidney transplant recipients reached the threshold during the first 3 months after transplantation.

With a top tercile cutoff of 7% abundance, a high relative abundance of *Faecalibacterium* was significantly associated with a lower risk of development of *Enterobacteriaceae* bacteriuria (Hazard ratio [HR] 0.3, 95% confidence interval [CI] 0.1–0.7, *P* = .01). With a top tercile cutoff of 7% abundance, a high relative abundance of *Romboutsia* was not significantly associated with a lower risk of development of *Enterobacteriacaeae* bacteriuria (HR 0.8, 95% CI 0.4–1.5, *P* = .43). With a top tercile cutoff of 1.6%, a high relative abundance of *Lactobacillus* was significantly associated with an increased risk of development of *Enterobacteriaceae* bacteriuria (HR 3.0, 95% CI 1.7–5.4, *P* < .001). With a top tercile cutoff of 13.7%, a high combined relative abundance of *Faecalibacterium* and *Romboutsia* was significantly associated with a lower risk of future development of *Enterobacteriaceae* bacteriuria (HR 0.2, 95% CI 0.1–0.7, *P* = .008).

With a top tercile cutoff of 7% abundance, a high relative abundance of *Faecalibacterium* was not significantly associated with a lower risk of future development of *Enterobacteriaceae* UTI (HR 0.6, 95% CI 0.2–1.5, *P* = .23). With a top tercile cutoff of 7% abundance, a high relative abundance of *Romboutsia* was not significantly associated with a lower risk of development of *Enterobacteriacaeae* UTI (HR 0.8, 95% CI 0.3–1.8, *P* = .53). With a top tercile cutoff of 1.6%, a high relative abundance of *Lactobacillus* was significantly associated with an increased risk of development of *Enterobacteriaceae* UTI (HR 3.0, 95% CI 1.5–6.0, *P* = .002). However, with a top tercile cutoff of 13.7%, a high combined relative abundance of *Faecalibacterium* and *Romboutsia* was significantly associated with a lower risk of future development of *Enterobacteriaceae* UTI (HR 0.3, 95% CI 0.1–0.9, *P* = .04).

In order to account for significant risk factors such as gender and cefazolin antibiotic prophylaxis, we performed a multivariable cox regression analysis for the development of *Enterobacteriaceae* bacteriuria and UTI with the combined relative abundance of *Faecalibacterium* and *Romboutsia* as a time-dependent covariate and first antibiotic administration as a time dependent covariate (Table 3). In multivariate analysis, the combined relative abundance of *Faecalibacterium* and *Romboutsia* of greater than 13.7% was significantly associated with future development of *Enterobacteriaceae* bacteriuria (HR 0.3, 95% CI 0.1–0.8, *P* = .02) (Table 3A) and future development of *Enterobacteriaceae* UTI (HR 0.4, 95% CI 0.1–1.2, *P* = .09) (Table 3B). We also evaluated the relative abundance of *Lactobacillus* as a time-dependent covariate and first antibiotic administration as a time dependent covariate. In multivariate analysis, the relative abundance of *Lactobacillus* of greater than 1.6% was significantly associated with future development of *Enterobacteriaceae* bacteriuria (HR 2.4, 95% CI 1.3–4.2, *P* = .003) and future development of *Enterobacteriaceae* UTI (HR 2.3, 95% CI 1.1–4.7, *P* = .02) (Supplemental Table 1).

**Table 2.** **Comparison of the Most Abundant Genera between the *Enterobacteriaceae* Bacteriuria Group and the No *Enterobactericaeae* Bacteriuria Group**. For each of the top 10 genera, the relative abundance of 153 fecal specimens from 51 patients in the *Enterobacteriaceae* Bacteriuria Group was compared to the relative abundance of 357 fecal specimens from the 117 patients in the No *Enterobacteriaceae* Bacteriuria Group using a Wilcoxon rank sum test. The adjusted *P* values were calculated using a Bonferonni correction. In bold are the genera that were significantly different between the groups (Adjusted *P* < .01).

	No Enterobacteriaceae	Enterobacteriaceae		
	Bacteriuria Group	Bacteriuria Group		
	(357 specimens,	(153 specimens,		
	117 subjects)	51 subjects)		
	Median Gut Relative	Median Gut Relative		
Genus	Abundance (%)	Abundance (%)	P value	Adjusted *P* value
***Lactobacillus***	**0.04%**	**0.21%**	**1.0 x 10^–7^**	**1.0 x 10^−6^**
***Faecalibacterium***	**0.80%**	**0.03%**	**1.4 x 10^–7^**	**1.4 x 10^−6^**
***Romboutsia***	**0.39%**	**0.06%**	**3.4 x 10^–4^**	**0.003**
*Blautia*	17.07%	13.25%	0.001	0.013
*Ruminococcus*	2.45%	1.59%	0.011	0.108
*Streptococcus*	0.64%	1.28%	0.016	0.162
*Ruminiclostridium*	1.45%	0.97%	0.038	0.376
*Gemmiger*	1.84%	0.33%	0.062	0.621
*Bifidobacterium*	0.27%	0.27%	0.538	0.999
*Erysipelatoclostridium*	5.45%	4.68%	0.699	0.999

**Table 3.** **Multivariable Cox Regression for *Enterobacteriaceae* Bacteriuria and *Enterobacteriaceae* UTI**. Univariate Cox regression analysis was performed for each of the characteristics and the development of *Enterobacteriaceae* bacteriuria or *Enterobacteriaceae* UTI. The combined relative abundance of *Faecalibacterium* and *Romboutsia* (cutoff of 13.8%) was analyzed as a time-dependent covariate and first antibiotic administration was analyzed as a time-dependent covariate. For characteristics that were significantly associated with either *Enterobacteriaceae* bacteriuria or *Enterobacteriaceae* UTI (*P* < .10), a multivariable Cox Regression was performed with the significantly associated characteristics. Table 3A. Multivariable Cox Regression for *Enterobacteriaceae* bacteriuria. Table 3B. Multivariable Cox Regression for *Enterobacteriaceae* UTI. ESRD, end stage renal disease; DM, diabetes mellitus; HTN, hypertension; PRA panel reactive antibody; PCP, *Pneumocystis jiroveci.*

Table 3A								
Risk Factors for *Enterobacteriaceae* UTI		Univariate Analysis			Multivariate Analysis			
Characteristic		HR (95% CI)	P value		HR (95% CI)		P value	
Age, Years		1.0 (1.0–1.0)	0.54					
Female Gender		4.1 (2.2–7.6)	6.8 x 10^−6^		4.4 (2.3–8.2)		3.9 x 10^−6^	
African American Race		0.9 (0.4–1.6)	0.65					
History of Diabetes Mellitus		1.4 (0.8–2.4)	0.29					
Cause of ESRD – DM		1.4 (0.8–2.5)	0.29					
Cause of ESRD – HTN		0.9 (0.4–2.1)	0.82					
PRA ≥ 80%		1.1 (0.4–2.8)	0.81					
Decreased Donor Transplantation		1.2 (0.7–2.2)	0.49					
Delayed Graft Function		1.2 (0.6–2.5)	0.53					
Cefazolin Preoperative Abx		0.5 (0.2–0.9)	0.01		0.5 (0.2–0.9)		0.02	
Trimethoprim/Sulfamethoxazole PCP Prophylaxis		3.2 (0.4–23.2)	0.25					
Anti-thymocyte Globulin Induction		1.1 (0.6–2.1)	0.82					
Prednisone Maintenance		1.3 (0.7–2.4)	0.34					
First Antibiotic Administration		2.1 (1.1–3.8)	0.02		1.4 (0.7–2.6)		0.33	
Relative Abundance of *Faecalibacterium* & *Romboutsia*		0.2 (0.1–0.7)	0.008		0.3 (0.1–0.8)		0.02	
Table 3B								
Risk Factors for *Enterobacteriaceae* UTI		Univariate Analysis			Multivariate Analysis			
Characteristic		HR (95% CI)	P value		HR (95% CI)		P value	
Age, Years		1.0 (1.0–1.0)	0.31					
Female Gender		4.4 (2.0–9.7)	2.8 x 10^–4^		4.4 (2.0–9.9)		3.2 x 10^–4^	
African American Race		0.8 (0.3–1.8)	0.55					
History of Diabetes Mellitus		1.3 (0.7–2.7)	0.44					
Cause of ESRD – DM		1.2 (0.6–2.5)	0.60					
Cause of ESRD – HTN		0.5 (0.2–1.9)	0.33					
PRA ≥ 80%		0.7 (0.2–3.0)	0.63					
Decreased Donor Transplantation		1.1 (0.5–2.2)	0.88					
Delayed Graft Function		0.9 (0.3–2.3)	0.80					
Cefazolin Preoperative Abx		0.5 (0.2–1.1)	0.07		0.5 (0.2–1.2)		0.12	
Trimethoprim/Sulfamethoxazole PCP Prophylaxis		– –	– –					
Anti-thymocyte Globulin Induction		1.0 (0.5–2.3)	0.95					
Prednisone Maintenance		1.0 (0.5–2.1)	0.99					
First Antibiotic Administration		2.2 (1.1–4.7)	0.03		1.5 (0.7–3.3)		0.29	
Relative Abundance of *Faecalibacterium* & *Romboutsia*		0.3 (0.1–0.9)	0.04		0.4 (0.1–1.2)		0.09	

## Discussion

In this study, we identified that high relative abundances of two taxa, *Faecalibacterium* and *Romboutsia*, are associated with a decreased risk for *Enterobacteriaceae* bacteriuria and UTI in kidney transplant recipients. We further report an inverse relationship of the relative abundances of these two taxa with the relative abundance of *Enterobacteriaceae*. These data provide further support for a growing notion that gut commensal organisms are associated with lower risk of infectious complications, which is well-established for *Clostridioides difficile* disease.

Recent studies have shown that the relative abundance of pathogenic bacteria is associated with UTI development. Thanert and colleagues evaluated 14 non-transplant patients with recurrent UTIs or non-recurrent UTIs and found that the gut was one of three different sources for recurrence of UTIs.^11^ In a study that we performed at our center, we observed that the gut abundance of *Escherichia* was associated with future development of *Escherichia* bacteriuria and UTI.^9^ These studies investigated the relationship between the relative abundance of pathogenic bacteria and UTI development. The current study is the first, to the best of our knowledge, to investigate whether high relative abundance of commensal organisms is associated with lower rates of UTI development.

Rising rates of multidrug resistant uropathogens and stagnating development of novel antimicrobials^17^ have led to an increased need for novel therapies to treat UTIs. Modulation of the gut microbiota via FMT constitute one major new line of research. In a study of 8 non-transplant patients with recurrent *C. difficile* infections, FMT was associated with a significant decrease in UTI recurrence.^14^ In a case of a heart and kidney transplant recipient who had recurrent vancomycin-resistant *Enterococcus* (VRE) bacteremia and UTIs, FMT was performed because of recurrent *C. difficile* infection and was associated with resolution of the VRE infections.^18^ In another case, FMT was performed in a kidney transplant recipient who had recurrent multidrug resistant *K. pneumoniae* UTIs, which was associated with eradication of the multidrug resistant *K. pneumoniae* and resolution of *K. pneumoniae* UTIs.^13^ However, FMT is largely an untargeted approach and most recently has been associated with lethal complications in immunocompromised patients.^19^ Thus there is a need to identify commensal bacterial taxa that could confer protection against pathogenic bacteria. Our study’s identification of higher relative abundance of *Faecalibacterium* and *Romboutsia* as being associated with decreased risk for *Enterobacteriaceae* UTI may thus help to better personalize the use of FMT for patients with recurrent *Enterobacteriaceae* UTIs. It could also lead to development of personalized consortia of probiotics for the prevention of UTIs.

Importantly, we also found an inverse correlation between the relative abundances of *Faecalibacterium* and *Romboutsia* and the relative abundance of *Enterobacteriaceae*. In this study, we did not test this relationship *in vitro* so we do not know the mechanism for this inverse relationship in our cohort. We speculate that one of the mechanisms by which low relative abundances of *Faecalibacterium* or *Romboutsia* could inhibit growth of *Enterobacteriaceae* is through short-chain fatty acid (SCFA) production. An elegant study by the Pamer group found that a mixture of short-chain fatty acids (SCFAs) and acidic pH inhibited the growth of *E. coli* and *K. pneumoniae in vivo* and that their growth is inhibited because of intracellular acidification from SCFAs.^20^ *Faecalibacterium* has been described as one of the most abundant and important producers of the SCFA butyrate in the intestine.^21^ Species in the *Romboutsia* taxa are also predicted to be associated with production of the SCFA acetate.^22^ It is plausible that even though the relative abundances of *Faecalibacterium* or *Romboutsia* are low, these bacteria produce sufficient SCFAs to have protective effects on the growth and relative abundances of *Enterobacteriaceae.*

In this study, we also found that antibiotic administration was associated with decreased relative abundance of *Faecalibacterium* and decreased relative abundance of *Romboutsia*. While antibiotics have been described to reduce various commensal bacterial taxa,^15,16^ our study highlights the effects of antibiotics on these two taxa that we have identified as associated with future development of *Enterobacteriaceae* bacteriuria and UTI. Our data suggests that antibiotic administration could lead to the development of infections beyond *C. difficile* disease. Interestingly, female kidney transplant recipients had lower relative abundance of *Faecalibacterium* and *Romboutsia* and higher relative abundance of *Lactobacillus* than male transplant recipients. Importantly, we controlled for gender in the Cox Regression analysis for development of *Enterobacteriaceae* bacteriuria and UTI as female gender is independently associated with development of UTI in kidney transplant recipients.^4^ While female gender was a significant risk factor for *Enterobacteriaceae* bacteriuria and UTI, the relative abundance of the identified taxa were also independently associated with development of the outcomes.

Interestingly, we also report that the relative abundance of *Lactobacillus* is associated with an increased hazard ratio for the development of *Enterobacteriaceae* bacteriuria and UTI. With respect to microbial interactions with *Lactobacillus*, we have found that the relative abundance of *Lactobacillus* is correlated with decreased microbial diversity, is negatively correlated with the combined relative abundance of *Faecalibacterium* and *Romboutsia*, but not correlated with the relative abundance of *Enterobacteriaceae*. Vaginal suppositories of *Lactobacillus crispatus* has been associated with decreased development of UTI ^23^ and mixed data exists on the use of oral *Lactobacillus* species for the prevention of UTI.^24^ It is possible that the gut abundance of *Lactobacillus* does not directly correlate with vaginal or urinary abundances of *Lactobacillus*. Furthermore, from our correlational data, *Lactobacillus* could be a biomarker for decreased microbial diversity manifested by decreased relative abundance of commensal bacterial taxa such as *Faecalibacterium* and *Romboutsia*.

There are several limitations to our study. While we had comprehensive evaluation of bacteriuria development given that kidney transplant recipients provided urine specimens at every clinical outpatient visit, we retrospectively reviewed the medical charts to identify UTI symptoms. It is possible that some patients did not have UTI symptoms recorded in the medical record and thus some UTIs could be misclassified as bacteriuria, which could explain discrepancies between the Cox Regression analyses for bacteriuria and UTI. However, given the critical clinical importance of documenting and treating UTIs in kidney transplant recipients, we believe that most symptomatic UTIs were captured. Diet has also been shown to change the gut microbiota^25^ and we did not evaluate the effect of diet on the gut microbiota in the transplant recipients in this cohort. The use of probiotics such as *Lactobacillus* in the cohort was not obtained in the transplant population and reflects another limitation of the study.

In conclusion, we identified that high relative abundances of *Faecalibacterium* and *Romboutsia* are associated with decreased risk for *Enterobacteriaceae* bacteriuria and UTI development in kidney transplant recipients. Our data support future studies evaluating the use of gut microbial based therapies for the prevention of recurrent UTIs in the kidney transplant population, which may be further generalizable to non-transplant patients with recurrent UTIs.

## Patients and methods

### Kidney transplant cohort

One hundred sixty-eight kidney transplant recipients who had kidney transplantations from August 2015 to November 2016 at NewYork-Presbyterian Hospital – Weill Cornell Medical Center were recruited to provide serial fecal specimens in the first 3 months after transplantation. The Weill Cornell Medicine Institutional Review Board approved this study (IRB # 1207012730) and each of the patients provided written informed consent. Kidney transplant recipients at our center undergo routine microscopic urinalysis and conventional urine culture at every clinical visit (approximately twice weekly in the first month, weekly in the second month, every 2 weeks in the third month, and monthly in the fourth, fifth, and sixth month), providing a comprehensive evaluation of bacteriuria. Kidney transplant recipients were considered to have *Enterobacteriaceae* bacteriuria if they had a positive urine culture (≥10,000 colony-forming units of a species from the *Enterobacteriaceae* family per mL of urine) during the first 6 months after transplantation and to have *Enterobacteriaceae* UTI if they had *Enterobacteriaceae* bacteriuria and symptoms of dysuria, frequency, urgency, or fever^26^ during the first 6 months after transplantation. Urine specimens were analyzed according to standard of care procedures at NewYork Presbyterian Hospital – Weill Cornell Medical Center clinical microbiology laboratory. Kidney transplant recipients receive surgical pre-operative antibiotic prophylaxis (most commonly cefazolin) and *Pneumocystis jiroveci* prophylaxis (most commonly trimethoprim-sulfamethoxazole).

### Fecal specimen collections

Patients were instructed to collect fecal specimens using a Fisherbrand toilet specimen collection kit (Fisher Scientific, New Hampton, NH, USA). The fecal specimen was aliquoted and stored at −80°C. Patients were asked to collect fecal specimens at post-transplant week 1, week 2, week 4, week 12, and during episodes of UTIs and diarrhea.

### DNA extraction and 16S rRNA gene sequencing

DNA extraction, 16S rRNA gene amplification, and deep sequencing of the 16S rRNA amplicon were previously performed and complete details of the protocol can be found in Magruder et al.^9^ In brief, DNA was isolated using a bead-beater phenol-chloroform extraction method. The V4-V5 region of the 16S rRNA gene (563 F and 926 R) was amplified with barcodes for multiplexing. The PCR amplicons were quantified and pooled. The Illumina TruSeq Sample Preparation protocol was used to add Illumina barcodes and adaptors (Illumina Inc., San Diego, CA, USA). The products were sequenced on an Illumina MiSeq Instrument (250 base pair by 250 base pair).

### Bioinformatics and statistical analyses

Details of the bioinformatics to assign taxonomy are found in Magruder et al. In brief, the 16S rRNA gene paired-end reads were merged and demultiplexed. The UPARSE pipeline^27^ was used to group sequences into operational taxonomic units by 97% distance-based similarity and remove potential chimeric sequences. Taxonomic assignment was done using nucleotide BLAST^28^ with NCBI RefSeq^29^ as the reference training set with a minimum E-value threshold of 10^−10^. The distribution of categorical values was analyzed using two-tailed Fisher’s exact test and the distribution of continuous variables was compared using the two-tailed Wilcoxon rank sum test. Correlation between two continuous variables was analyzed using a Pearson’s correlation. A Cox Regression Hazard Model was utilized to assess the relationship between the relative abundance of specific taxa and the development of bacteriuria or UTI. In this model, the relative abundance of the specific taxa was a time-ever dependent covariate where it was assumed that the relative abundance was not crossed until the first time the relative abundance value crossed the threshold. A multivariable Cox Regression was performing including significant clinical variables. All analyses were performed in R 3.3.3.

## Supplementary Material

Supplemental Material

## Data Availability

All sequencing data and the deidentified clinical data will be made available upon publication at accession number phs001879 in the database of Genotypes and Phenotypes (dbGaP). Local institutional review board approval will be necessary to obtain the data.
