# Supplementary material for: Gut commensal microbiota and decreased risk for Enterobacteriaceae bacteriuria and urinary tract infection
Source: Gut Microbes. 2020 Aug 30;12(1):1805281. doi: 10.1080/19490976.2020.1805281 (PMC7524266; doi:10.1080/19490976.2020.1805281)
Supplement: Supplemental Material [file KGMI_A_1805281_SM0387.docx]

**Supplementary Information**

**Gut Commensal Microbiota and Decreased Risk for *Enterobacteriaceae* Bacteriuria and Urinary Tract Infection**

Matthew Magruder^1^, Emmanuel Edusei^1^, Lisa Zhang^1^, Shady Albakry^1^, Michael J Satlin^2^, Lars F Westblade^2,3^, Line Malha^1^, Christina Sze^4^, Michelle Lubetzky^1,5^, Darshana M. Dadhania^1,5^, John Richard Lee*^1,5^

^1^ Division of Nephrology and Hypertension, Department of Medicine, Weill Cornell Medicine, New York, NY

^2^ Division of Infectious Diseases, Department of Medicine, Weill Cornell Medicine, New York, NY

^3^ Department of Pathology and Laboratory Medicine, Weill Cornell Medicine, New York, NY

^4^ Department of Urology, NewYork Presbyterian Hospital – Weill Cornell Medical Center, New York, NY

^5^ Department of Transplantation Medicine, New York Presbyterian Hospital – Weill Cornell Medical Center, New York, NY

* Address correspondence to: Dr. John Richard Lee, jrl2002@med.cornell.edu

Short Title: Gut Commensal Microbiota and Urinary Tract Infection

Key Words: Microbiota, Bacteriuria, Urinary Tract Infection, *Enterobacteriaceae, Faecalibacterium, Romboutsia, Lactobacillus*

**Supplemental Table 1**

**
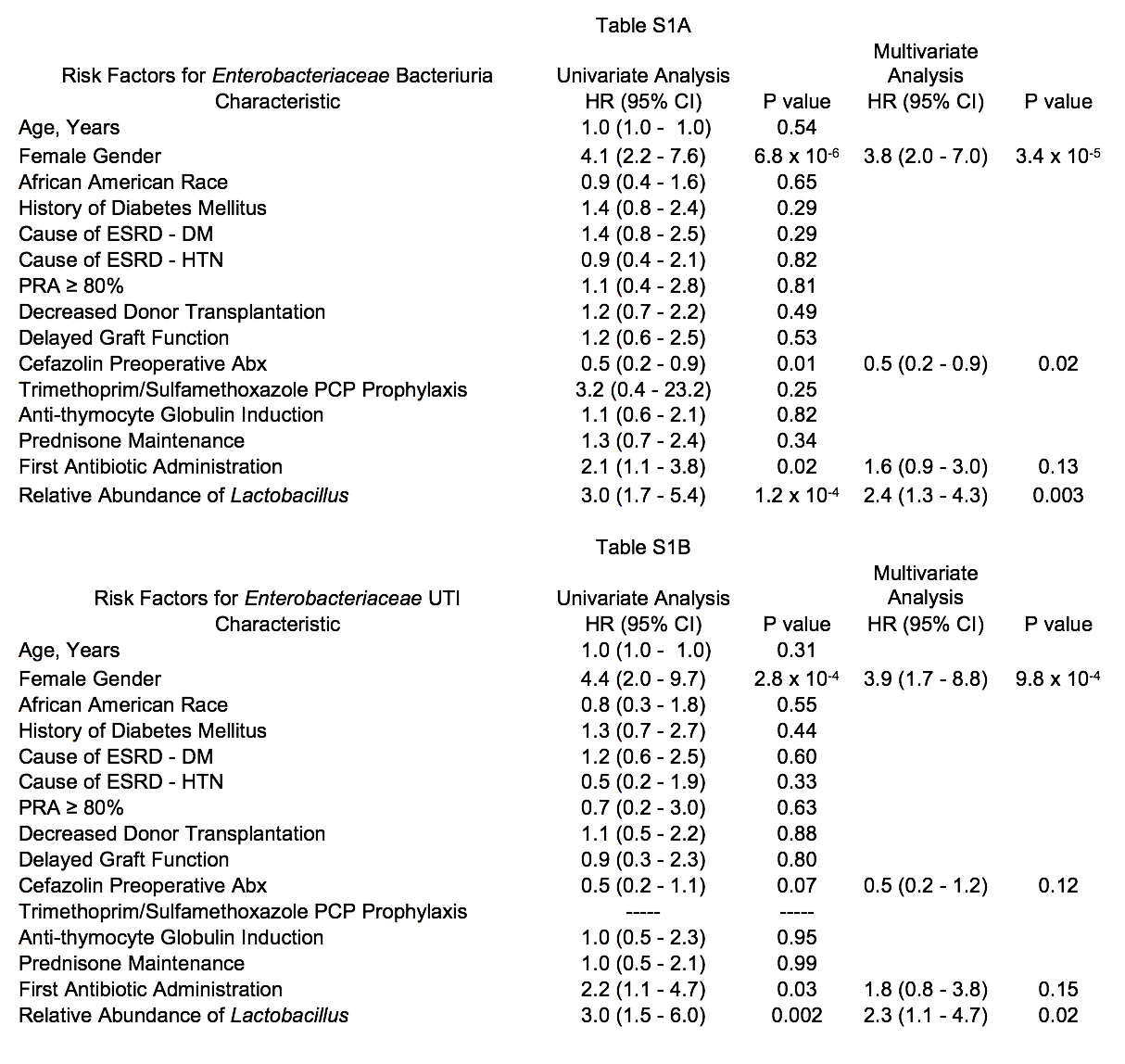
**

**Supplemental Table 1. Multivariable Cox Regression for *Enterobacteriaceae* Bacteriuria and *Enterobacteriaceae* UTI.** Univariate Cox regression analysis was performed for each of the characteristics and the development of *Enterobacteriaceae* bacteriuria or *Enterobacteriaceae* UTI. The relative abundance of *Lactobacillus* (cutoff of 1.6%) was analyzed as a time-dependent covariate and first antibiotic administration was analyzed as a time-dependent covariate. For characteristics that were significantly associated with either *Enterobacteriaceae* bacteriuria or *Enterobacteriaceae* UTI (P < 0.10), a multivariable Cox Regression was performed with the significantly associated characteristics. Table S1A. Multivariable Cox Regression for *Enterobacteriaceae* bacteriuria. Table S1B. Multivariable Cox Regression for *Enterobacteriaceae* UTI. ESRD, end stage renal disease; DM, diabetes mellitus; HTN, hypertension; PRA panel reactive antibody; PCP, *Pneumocystis jiroveci*

**Supplementary Figure 1**

**
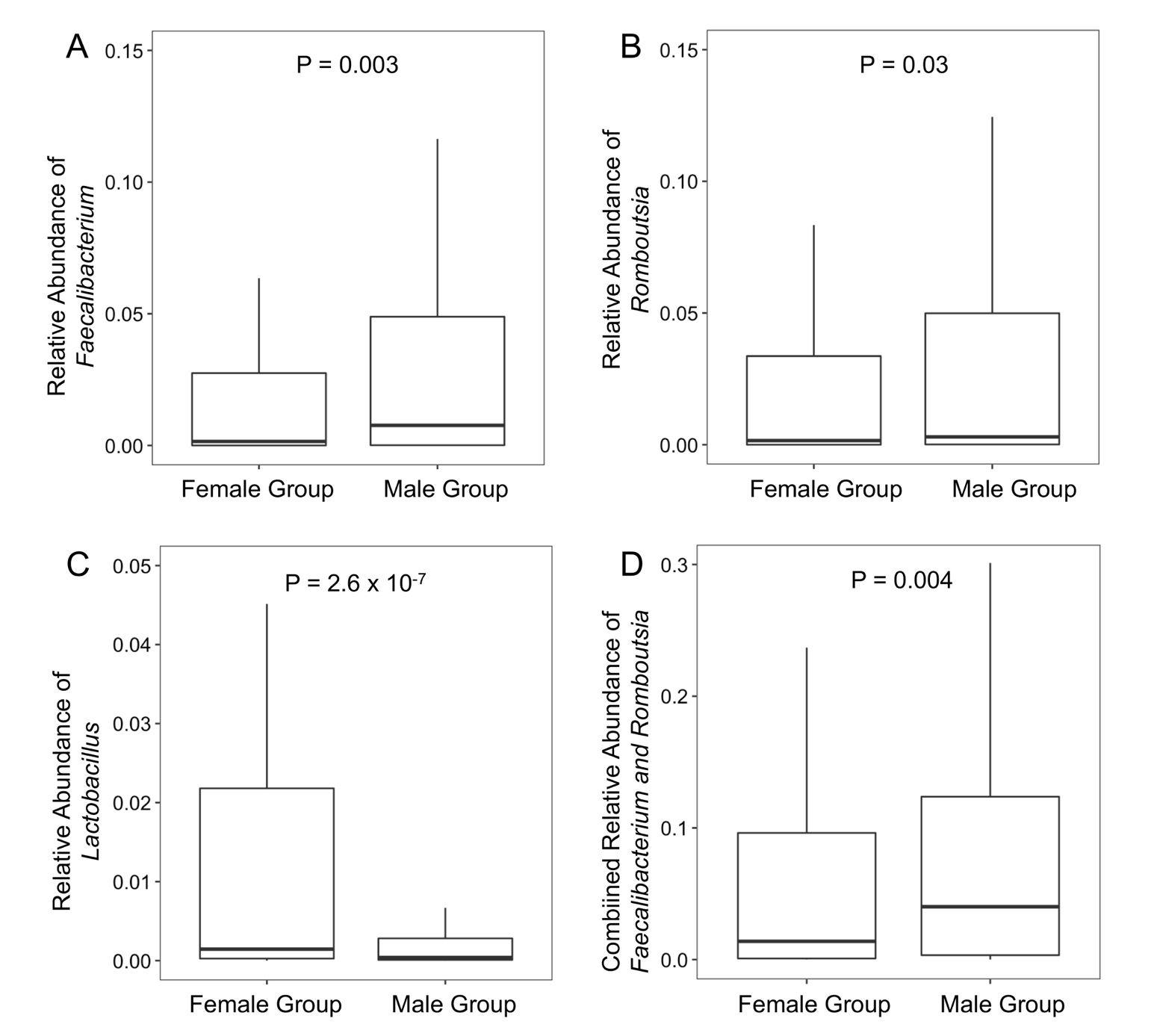
**

**Supplementary Figure 1. Relative abundances of *Faecalibacterium, Romboutsia,* and *Lactobacillus* by gender.** Box and whisker plots are represented in each graph with the relative abundance of taxa on the y axis and gender on the x axis. The line in the box plot represents the median; the box represents the 25^th^ and 75^th^ percentile values; and the whiskers represent the farthest value of 1.5 times the interquartile range. There were 76 female patients who contributed 224 fecal specimens and 92 male patients who contributed 286 fecal specimens. P value was calculated using the Wilcoxon rank sum test. **Panel A.** Relative abundance of *Faecalibacterium.* **Panel B.** Relative abundance of *Romboutsia.* **Panel C.** Relative abundance of *Lactobacillus****.* Panel D.** Combined relative abundance of *Faecalibacterium and Romboutsia.*

**Supplementary Figure 2**

**
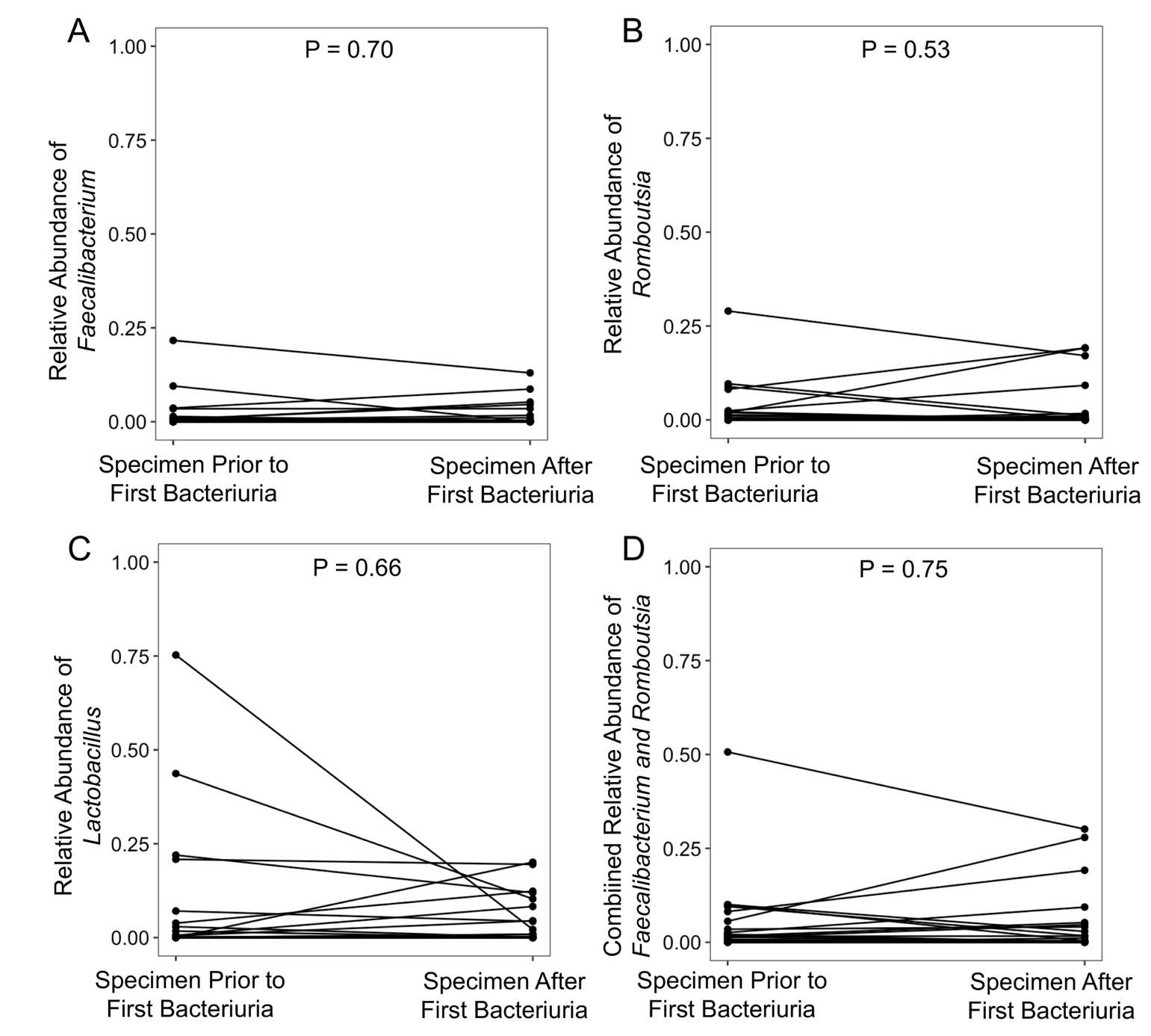
**

**Supplementary Figure 2. Changes in the relative abundances of *Faecalibacterium, Romboutsia,* and *Lactobacillus* before and after *Enterobacteriaceae* bacteriuria.** There were 24 patients who had a fecal specimen prior to the diagnosis of first *Enterobacteriaceae* bacteriuria and a fecal specimen closest after the diagnosis of first *Enterobacteriaceae* bacteriuria. Each point represents a fecal specimen and the line connecting points represent an individual patient before and after first *Enterobacteriaceae* bacteriuria. P value was calculated using the Wilcoxon signed-rank test. **Panel A.** Relative abundance of *Faecalibacterium.* **Panel B.** Relative abundance of *Romboutsia.* **Panel C.** Relative abundance of *Lactobacillus****.* Panel D.** Combined relative abundance of *Faecalibacterium and Romboutsia.*

**Supplementary Figure 3**

**
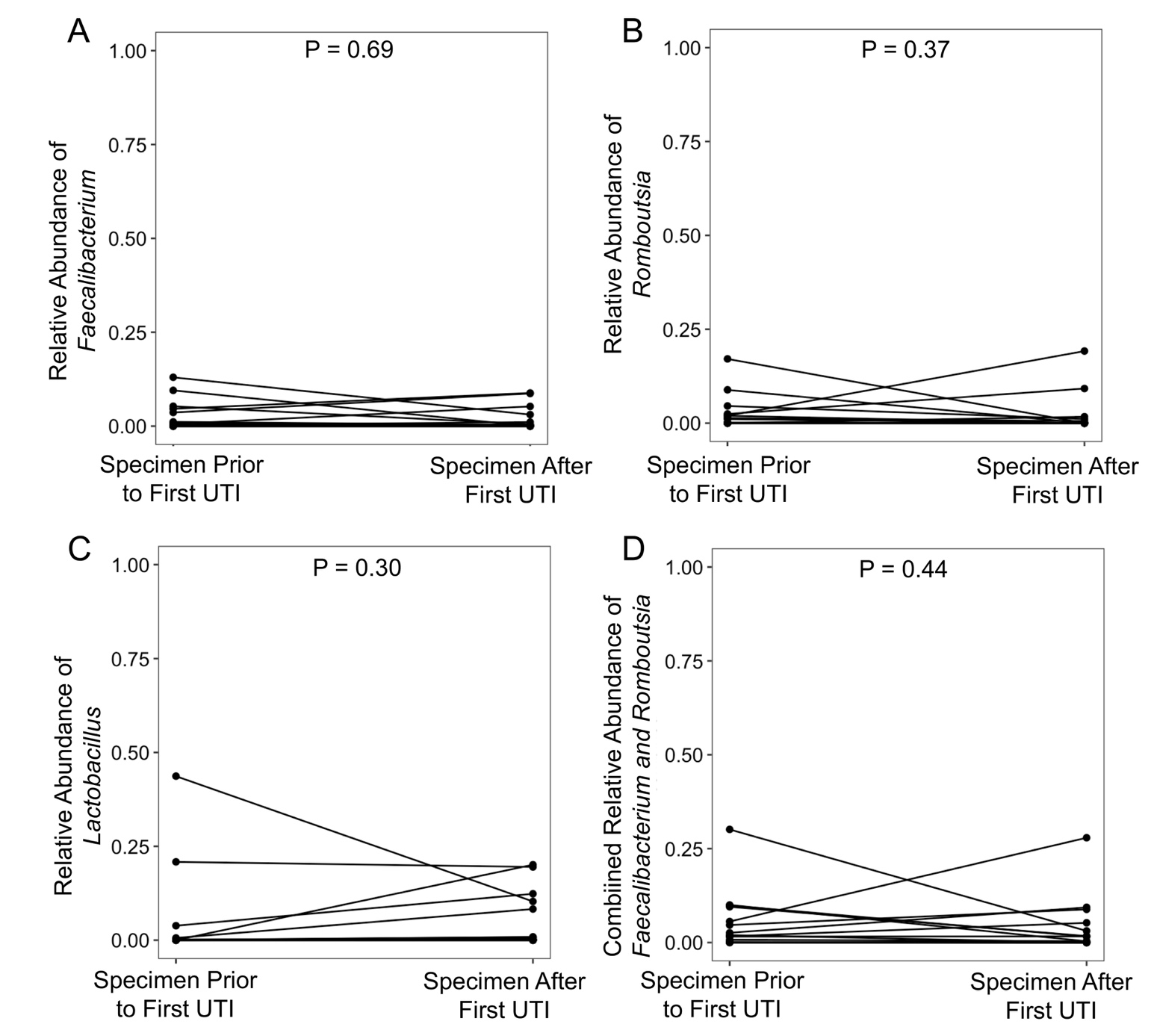
**

**Supplementary Figure 3. Changes in the relative abundances of *Faecalibacterium, Romboutsia,* and *Lactobacillus* before and after *Enterobacteriaceae* UTI.** There were 15 patients who had a fecal specimen prior to the diagnosis of first *Enterobacteriaceae* UTI and a fecal specimen closest after the diagnosis of first *Enterobacteriaceae* UTI. Each point represents a fecal specimen and the line connecting points represent an individual patient before and after first *Enterobacteriaceae* UTI. P value was calculated using the Wilcoxon signed-rank test. **Panel A.** Relative abundance of *Faecalibacterium.* **Panel B.** Relative abundance of *Romboutsia.* **Panel C.** Relative abundance of *Lactobacillus****.* Panel D.** Combined relative abundance of *Faecalibacterium and Romboutsia.*

**Supplemental Figure 4**

**
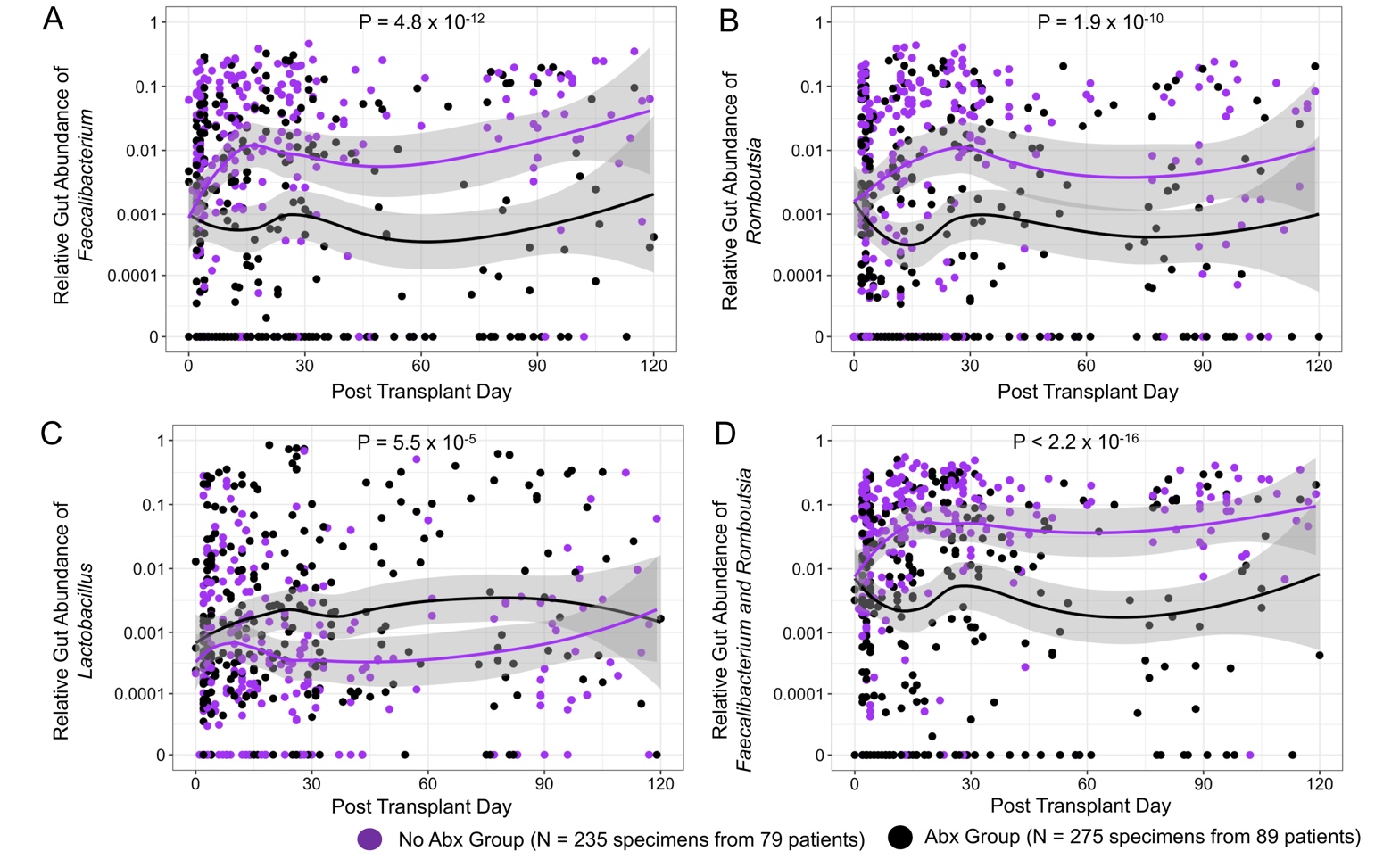
**

**Supplemental Figure 4. Relative Abundance of *Faecalibacterium, Romboutsia,* and *Lactobacillus* by Antibiotic Group Status.** The 510 fecal specimens are shown in each graph with the No Abx Group consisting of 235 fecal specimens from 79 patients and the Abx Group consisting of 275 fecal specimens from 89 patients. Each point represents a fecal specimen and the point’s color represents Antibiotic Group status. The relative abundance of genera is on the y axis (log_10_ scale) and the post-transplant day is on the x axis. The line represents a locally estimated scatterplot smoothing (LOESS) curve with 95% confidence intervals in the shaded area. P values were calculated using the Wilcoxon rank sum test. **Panel A.** Relative abundance of *Faecalibacterium.* **Panel B.** Relative abundance of *Romboutsia.* **Panel C.** Relative abundance of *Lactobacillus.* **Panel D.** Combined relative gut abundance of *Faecalibacterium* and *Romboutsia.*
